# Pharmacoepidemiology in pregnancy: analysis protocol for an observational cohort study in the UK Clinical Practice Research Datalink

**DOI:** 10.12688/wellcomeopenres.17523.1

**Published:** 2022-01-12

**Authors:** Ciarrah-Jane Barry, Christy Burden, Neil Davies, Venexia Walker

**Affiliations:** 1 Medical Research Council Integrative Epidemiology Unit, University of Bristol, Bristol, BS82BN, UK; 2 Population Health Sciences, Bristol Medical School, University of Bristol, Bristol, BS82BN, UK; 3 Translational Health Sciences, Bristol Medical School, University of Bristol, Bristol, UK; 4 K.G. Jebsen Center for Genetic Epidemiology, Department of Public Health and Nursing, Norwegian University of Science and Technology, Trondheim, Norway; 5 Department of Surgery, University of Pennsylvania Perelman School of Medicine, Philadelphia, Pennsylvania, USA

**Keywords:** UK Clinical Practice Research Datalink (CPRD), pharmacoepidemiology, intrauterine drug exposure, maternal health, prescriptive drug use neonatal health or outcomes

## Abstract

Large numbers of women take prescription and over-the-counter medications during pregnancy. However, there is very little definitive evidence about the potential effects of these drugs on the mothers and offspring. We will investigate the risks and benefits of continuing prescriptive drug use for chronic pre-existing maternal conditions such as diabetes, hypertension and thyroid related conditions throughout pregnancy. If left untreated, these conditions are established risk factors for adverse neonatal and maternal outcomes. However, some treatments for these conditions are associated with adverse neonatal outcomes.

Our primary aims are twofold. Firstly, we aim to estimate the beneficial effect on the mother of continuing treatment during pregnancy. Second, we aim to determine whether there is an associated detrimental impact on the neonate of continuation of maternal treatment during pregnancy. To establish this evidence, we will investigate the relationship between maternal drug prescriptions and adverse and beneficial offspring outcomes to provide evidence to guide clinical decisions.

We will conduct a hypothesis testing observational intergenerational cohort study using data from the UK Clinical Practice Research Datalink (CPRD). We will apply four statistical methods: multivariable adjusted regression, propensity score regression, instrumental variables analysis and negative control analysis. These methods should account for potential confounding when estimating the association between the drug exposure and maternal or neonatal outcome.

In this protocol we describe the aims, motivation, study design, cohort and statistical analyses of our study to aid reproducibility and transparency within research.

## Introduction

 Prescribing medication provides a challenge for physicians when considering a pregnant patient. Here the beneficial effect of the drug must be balanced against possible adverse effects on the mother and unborn child. For many drugs prescribed throughout pregnancy, there is very little definitive evidence of safety as pregnant women are rarely recruited to clinical trials for ethical and practical reasons ^1^. This presents a significant problem as it is increasingly common for pregnant women to be prescribed medication for chronic conditions that may precede or develop during the pregnancy ^2, 3^. Cardiovascular related conditions are relatively common, with up to 10% of pregnant women experiencing hypertension ^4, 5^. Although less common, endocrine conditions, such as diabetes and thyroid disorders, are persistent and problematic within pregnancy, affecting around 4% and 10% of pregnant women respectively ^6– 8^. These conditions are well-established risk factors for numerous adverse maternal and neonatal such as preeclampsia, stroke, and preterm birth and stillbirth or perinatal death if left untreated ^9– 11^. Yet, treatments for these conditions are associated with additional neonatal risks, with many studies finding evidence of teratogenic effects ^12– 15^. With limited pharmacoepidemiological data to support their effects in human populations, information to make clinical recommendations during pregnancy is limited.

This necessitates a method to safely and accurately discern whether there are adverse offspring outcomes associated with these treatments, alongside evaluating the extent of the maternal benefit. Electronic healthcare databases are a source of routinely collected, anonymised healthcare data which can be used to ethically investigate associations between intrauterine drug exposure and maternal and neonatal outcomes, whilst avoiding the possible dangers of implementing randomised control trials ^16^.

Here we use available observational electronic healthcare record data to investigate potential risks currently associated with prescriptive drugs under circumstances where we are unable to perform a randomised controlled trial. To establish this evidence, we will investigate the relationship between maternal drug prescriptions and adverse and beneficial offspring outcomes to provide evidence to guide clinical decisions.

## Methods

### Study design & data source

We will conduct an intergenerational hypothesis testing cohort study of maternal prescriptive drug use on maternal and neonatal outcomes, using data from the Clinical Practice Research Datalink (CPRD).

### Study population

We define two cohorts within our study population. To study maternal outcomes, we define cohort 1 as the following. Eligible patients must be on the CPRD Pregnancy Register at any time and be classified as ‘acceptable’ patients with a minimum of 12 months “up-to-standard’ practice prior to the estimated start of pregnancy, as defined on the CPRD Pregnancy Register ^16, 17^. Patients registered with less than 12 months data will be excluded to ensure sufficient quality baseline and covariate data. Patients must be registered with the practice at least until the end of the pregnancy, as defined by the Pregnancy register. In addition, patients must have a diagnosis of at least one condition of interest at least 6 months prior to the start of pregnancy, as identified through medical codes according to their Clinical file ^18^. To study neonatal outcomes, we define cohort 2 as the neonates of the eligible patients in cohort 1.

The above cohorts will be categorised based on exposure status to prescriptive medications during pregnancy for their respective conditions and their history of the condition, product code lists available in the extended data ^18^.

### Variables

Using the CPRD, an individual’s primary patient care data from can be linked to a range of datasets including Hospital Episode Statistics (HES), Death registration data from the Office for National Statistics (ONS), Index of Multiple deprivation (IMD), for a subset of patients ^19^. The CPRD has also developed an algorithm to link likely mother-offspring pairs within the primary care data, thus forming a Mother-Baby linkage dataset, and an additional algorithm was developed in 2019 to generated the Pregnancy Register data linkage ^17^. These linkages increase the quantity and depth of available information for individual patients. Linkages are available for approximately 58% of practices within the CPRD ^16^.

Maternal diagnoses and prescriptive events were available through Primary care data. Pregnancy related variables, such as the start and end of pregnancy, neonatal outcomes and birth information were available through the Mother-baby link and Pregnancy register.

### Exposure definition

We define the exposure to treatment based on the last prescriptions received before the pregnancy start date and the subsequent prescription after the establishment of pregnancy. Exposed women will be those prescribed medication of the same drug class (irrespective of brand, dose, formulation, or quantity) for the condition’s diabetes, hypertension or hyper- or hypothyroidism at least twice at any time from 6 months before the estimated conception date and at least once during their respective pregnancy as exposed.

Unexposed patients are be classified as those with an indication for the medication but not in receipt of at least two prescriptions prior to pregnancy and once during pregnancy; receipt of a prescription for one of the eligible medications, but too few prescriptions according to these criteria will also be counted as unexposed. We will use defined daily dose to obtain standardised estimates between drugs with varying dosages and regimes. Where individuals have been diagnosed with more than one relevant condition or have a prescription for more than one type of drug, they will be counted independently (i.e., each patient can have multiple comorbidities and prior treatment).

### Outcome definition

We define maternal outcomes via the clinical records from the Primary care, the Pregnancy register, HES and ONS data. Here we are investigating the effectiveness treatment on the mother during the pregnancy, Thus, maternal outcomes of interest include mode of delivery, gestational diabetes, episodes of hypo- or hyper-glycaemia antenatal or intrapartum, hypertensive disorders of pregnancy, post-natal diagnosis of severe hypertension, post-natal incidence of thyroid disorders, postpartum haemorrhage (PPH), urinary tract infection (ITU admission) and mortality.

We define adverse neonatal outcomes via the Pregnancy Register, and HES and ONS data and Read codes from CPRD clinical records. Here we are investigating the association of adverse neonatal outcomes with intrauterine exposure to prescriptive drug use. Hence neonatal outcomes of interest are stillbirth, miscarriage, birthweight, birth head circumferences, premature birth (< 37 weeks), gestation at birth, Apgar score <7, admission to neonatal unit, neonatal death, and congenital defects. Neonatal adverse events will be categorised as occurring at birth in this study.

### Covariate definition

There are noted differences in drug prescriptions likely influenced by sociodemographic characteristics and other comorbidities ^20^. Hence, we will include and control for the following covariates: maternal age, body mass index, region of residence, maternal smoking, maternal alcohol intake, consultation rate, marital status, socioeconomic measures (e.g., the index of multiple deprivation), parity and ethnicity. We will extract these from past CPRD clinical, additional and referral records prior to the index pregnancy (see extended data ^18^) including linked HES data. We will also control for the presence of additional conditions using the Cambridge multimorbidity index ^21^. Calendar year of delivery will be included as a control variable to evaluate how prescribed drugs for each condition have changed over time. Covariate missingness will be carefully assessed in each analysis and, if appropriate, we will apply multiple imputation.

We will determine health care utilization via the consultation rate, calculated by dividing the total number of clinic visits prior to the index date, divided by the length of patient follow up. We will investigate the impact of ascertainment bias by adjusting for consultation rate as a sensitivity analysis, as those who have more severe conditions are likely to have a higher consultation rate, therefore have greater opportunity for the diagnosis of chronic conditions.

## Statistical analysis plan

We will apply multiple statistical methods within this study, including multivariable adjusted regression, propensity score regression, instrumental variable analysis, and negative control analysis. Subsequently, we will assess the benefits and limitations of the applied analysis methods to determine the impact this may have on our inference.

1) Multivariable adjusted regression

We will use linear and logistic regression, as appropriate, to estimate the association between exposure to a drug of interest and events in the neonate or the mother. We will report the associations estimated for unadjusted and adjusted models, using the covariates listed above.

2) Propensity score regression

A further analysis we will perform to address possible confounding will be to an implementation of propensity score regression ^22, 23^. Here we will fit a model for the exposure (a binary measure of prescriptive drug use or not) and take the estimated exposure probabilities as the estimated propensity scores. This is akin to controlling for confounding as we may then match exposed and unexposed participants on propensity score and directly compare maternal and neonatal outcomes. We will then consider covariate balance and possible further adjustment if required. From this we may then determine the average treatment effect within the populations of interest.

3) Instrumental variable analysis

In addition, we will perform an instrumental variable analysis using physicians’ preference to continue treatment as an instrument for the exposure. As we cannot directly measure physicians’ preferences, we will instead use the prescription issued to their previous patient as a proxy for their preference. Where possible, we will use instruments based on multiple prior prescriptions to improve instrument strength and maximise power ^24– 26^. Hence, we will perform a secondary analysis implementing instrumental variables.

4) Negative control analysis

We will analyse maternal response to treatment using a negative control approach to determine the differential effects of drug exposure. A negative control is a tool to help identify non-causal associations between exposures and outcomes ^27^. In pregnancy pharmacoepidemiology, a negative control such as medication use prior to a pregnancy but not during, can help to identify whether any associations observed are causal (by in utero exposure to a drug and the association attenuates) or explained by another mechanism, such as confounding (the association does not attenuate).

### Sample size considerations

We first obtained feasibility counts for each condition of interest to this study to calculate the minimum detectable linear regression effect size. These counts were indicative of the sample sizes available within the CPRD as limited information is available at the feasibility count stage. Thus, feasibility count patients were those prescribed medication for hypertensive, glycaemic or thyroid conditions, defined as at least two prescriptions identified via Product codes in the Clinical files. Patients must also have at least one medical diagnosis of pregnancy (recorded in the CPRD Database). Both events occur before the end of the study period and occur within the ‘up-to-standard’ registration period. Patients must be female, aged between 11 and 49 at the study start, and have at least 12 months prior up-to-standard registration at study start.

A minimum of one-to-one matching is assumed within the study, thus number of unexposed match numbers of exposed for the purposes of these calculations. We assume a type 1 error of 0.5, minimum power of 0.8 and use the respective sample size from feasibility counts. Detectable linear regression effect sizes were determined assuming 5 predictors, thus adjusting for age, comorbidity index, exposure, BMI and parity using the ‘WebPower’ R package ^28^, Table 1.

**Table 1.** The minimum detectable effect size for continuous outcomes for each condition of interest, calculated from feasibility counts.

Condition of interest	Feasibility count (n)	Minimum detectable effect size
Glycaemic conditions	7,989	0.0016
Hypertension	142,609	0.0001
Thyroid conditions	15,942	0.0008

Binary maternal and neonatal outcomes may have lower power, we take the continuous calculations to be indicative. It is noted that the counts are expected to be conservative due to low numbers of pregnancies recorded in the CPRD in the study period. Hence, the power of this study will be considerable as it involves a large sample of data, thus we expect to determine effects even when they are relatively small.

## Study status

CPRD GOLD data has been extracted for analysis and is being validated and cleaned. A linkage request for the Pregnancy Register, HES, ONS Death registration data, IMD and Mother-Baby link has been made and is being processed by the CPRD.

## Dissemination of the study outcome

We aim to publish our findings to the academic community via peer reviewed publications and national and international conferences.

## Data availability

### Underlying data

The patient data in this study are provided by the Clinical Practice Research Datalink (CPRD) obtained under licence from the UK Medicines and Healthcare products Regulatory agency (MHRA). This data is only available upon approval of an application to the CPRD.

### Extended data

Figshare: CPRD_protocol_codelists.xlsx, https://doi.org/10.6084/m9.figshare.17299733 ^18^.

This project contains the following extended data:

- Product_dia.xlsx (CPRD diagnosis codelist for treatments of diabetes)
- Medical_dia.xlsx (CPRD Read codelist for diagnosis of diabetes)
- Product_hyt.xlsx (CPRD diagnosis codelist for treatments of hypertension)
- Medical_hyt.xlsx (CPRD Read codelist for diagnosis of hypertension)
- Product_thy.xlsx (CPRD diagnosis codelist for treatments of hyper- and hypothyroidism)
- Medical_thy.xlsx (CPRD Read codelist for diagnosis of hyper- and hypothyroidism)

Data are available under the terms of the Creative Commons Attribution 4.0 International license (CC-BY 4.0).

### Ethical approval

The study protocol was reviewed and approved by the Independent Scientific Advisory Committee (ISAC), an advisory body for the MHRA. The CPRD adheres to all UK and European laws and guidelines governing research. All data provided by the CPRD is anonymized.
