# Supplementary material for: Pharmacoepidemiology in pregnancy: analysis protocol for an observational cohort study in the UK Clinical Practice Research Datalink
Source: Wellcome Open Res. 2022 Jan 12;7:12. [Version 1] doi: 10.12688/wellcomeopenres.17523.1 (PMC10333778; doi:10.12688/wellcomeopenres.17523.1)
Supplement: Peer review history

# Peer review: Reviewer response for version 1

**DOI:** 10.21956/wellcomeopenres.19375.r60533

**Published:** 2023-07-10

**Authors:** Wing Chung Chang

**Affiliations:** 1 The University of Hong Kong, Hong Kong, Hong Kong

This is a study protocol of a pharmacoepidemiology in pregnancy, using UK CPRD electronic health record data, to examine the beneficial effect and risk of adverse maternal / neonatal outcomes of continuation of indicated treatment during pregnancy on chronic pre-existing maternal conditions (diabetes, hypertension, and thyroid diseases). The methodology is well-described and sound. The outcomes (maternal and neonatal outcomes) are well-defined, and a comprehensive array of covariates are included in the analyses to control for potential confounders. The study will also adopt various statistical approaches for analyses, including traditional multivariable adjusted regression analyses, and more advanced analyses of propensity-score regression (i.e., using propensity-score weighting for analysis), instrumental variable analysis, and negative control analysis (i.e., discontinued-medication group for comparison. 

 Overall, the study protocol is of good quality, the study objectives are clearly-defined and of significant clinical implications and the methodology is robust. 

 Minor issue: Given that some concurrent medication treatment during pregnancy may also increase risk of adverse maternal outcomes e.g. prenatal use of second-generation antipsychotics and gestational diabetes, SSRI and risk of PPH, pre-existing mental disorders and increased risk of adverse neonatal and maternal outcomes (e.g. schizophrenia, depression etc.), and past history of gestational DM, HT etc. Will this study consider to adequately address these potential confounders?

Is the study design appropriate for the research question?

Yes

Is the rationale for, and objectives of, the study clearly described?

Yes

Are sufficient details of the methods provided to allow replication by others?

Yes

Are the datasets clearly presented in a useable and accessible format?

Yes

Reviewer Expertise:

Psychiatric disorders; relationship of mental disorders and psychotropic exposure with adverse maternal and neonatal outcomes

I confirm that I have read this submission and believe that I have an appropriate level of expertise to confirm that it is of an acceptable scientific standard.

# Peer review: Reviewer response for version 1

**DOI:** 10.21956/wellcomeopenres.19375.r48701

**Published:** 2022-03-07

**Authors:** Daniel R. Morales

**Affiliations:** 1 Population Health and Genomics Division, University of Dundee, Dundee, UK; 2 Department of Public Health, University of Southern Denmark, Odense, Denmark

The protocol is well written and acceptable for its purpose. Due to the strong effect of confounding by indication, the proposed analyses (3) & (4) are a useful addition. Minor suggestions could be:

1. Specify whether CPRD GOLD, AURUM or both are being used.
2. Consider listing the drug exposures of interest in the 'exposure definition'.
3. For the PS regression, state whether the same types of regression models will be used as in analysis 1 once matched.
4. Consider providing further detail on (3) IV analysis to clarify the approach e.g. clarify what is meant by the 'prescription issued to their previous patient' in this specific pregnancy setting.

Is the study design appropriate for the research question?

Yes

Is the rationale for, and objectives of, the study clearly described?

Yes

Are sufficient details of the methods provided to allow replication by others?

Partly

Are the datasets clearly presented in a useable and accessible format?

Not applicable

Reviewer Expertise:

Epidemiology/Pharmacoepidemiology/Primary Care

I confirm that I have read this submission and believe that I have an appropriate level of expertise to confirm that it is of an acceptable scientific standard.
